# Beneficial effects of cranberry juice enriched with omega-3 fatty acids in patients with type 2 diabetic and periodontal disease: A randomized pilot clinical trial

**DOI:** 10.34172/japid.2024.019

**Published:** 2024-09-11

**Authors:** Elnaz Ashrafzadeh, Hossein Babaei, Maryam Ravanbakhsh, Ahmad Zare Javid, Leila Maghsoumi-Norouzabad

**Affiliations:** ^1^Nutrition and Metabolic Diseases Research Center & Hyperlipidemia Research Center, Ahvaz Jundishapur University of Medical Sciences, Ahvaz, Iran; ^2^Student Research Committee, Ahvaz Jundishapur University of Medical Sciences, Ahvaz, Iran; ^3^Department of Nutrition, School of Allied Medical Sciences, Ahvaz Jundishapur University of Medical Sciences, Ahvaz, Iran; ^4^Drug Applied Research Center, Tabriz University of Medical Sciences, Tabriz, Iran; ^5^Research Center for Integrative Medicine in Aging, Aging Institute, Tabriz University of Medical Sciences, Tabriz, Iran

**Keywords:** Cranberry, Inflammation, Omega-3 fatty acid, Oxidative stress, Periodontal disease, Type 2 diabetes mellitus

## Abstract

**Background.:**

The present study evaluated the effects of cranberry juice enriched with omega-3 on inflammatory, oxidative stress, and periodontal status in diabetic patients with periodontal disease.

**Methods.:**

Forty-one patients with diabetes (35‒67 years old) and periodontal disease were assigned to four groups: C: control (n=12), I1: omega-3 (n=10, 1 g), I2: cranberry juice (n=9, 200 mL), and I3: cranberry juice enriched with omega-3 (n=10, 200 mL, containing 1 g of omega-3) twice daily for 8 weeks. Serum and salivary total antioxidant capacity (TAC), malondialdehyde (MDA), serum uric acid, tumor necrosis factor-alpha (TNF-α), interleukin-6 (IL-6), high-sensitivity C-reactive protein (hs-CRP), clinical attachment loss (CAL), pocket depth (PD), bleeding on probing (BOP), and plaque index were evaluated in all the subjects before and after the intervention.

**Results.:**

Serum and salivary TAC increased, and salivary MDA decreased in the I3 group compared with the control group. Additionally, serum MDA decreased in the I2 and I3 groups while serum TAC increased. Serum hs-CRP, IL-6, and TNF-α decreased in the I3 group compared with the baseline. Furthermore, serum hs-CRP and IL-6 decreased in the I3 group compared with the control group. After the intervention, PD and CAL significantly reduced in all the groups.

**Conclusion.:**

The consumption of cranberry juice enriched with omega-3 can be helpful as adjuvant therapy with non-surgical periodontal treatment in decreasing serum levels of IL-6 and hs-CRP, as well as serum and salivary levels of MDA while also increasing serum and salivary levels of TAC.

## Introduction

 Type 2 diabetes mellitus and periodontal disease have a physiological relationship.^1^ Several experimental studies have addressed the mechanisms underlying the interaction between DM and periodontitis. In both periodontal diseases and DM, the major inflammatory markers with both local (periodontal destruction) and systemic (impaired glycemic control) effects are involved.^2,3^ It is indicated that in type 2 diabetic patients with periodontal disease, the levels of inflammatory markers such as C-reactive protein, IL-1β, TNF-α, and IL-6 are high, which may adversely affect blood glucose and lipid metabolism.^4^ The imbalance between reactive oxygen species (ROS) production and antioxidant defenses leads to oxidative stress. Oxidative stress is an important factor in developing periodontal disease and DM.^5^ The findings of the Thomas et al study indicate that serum TAC was greater in the systemically healthy group without periodontitis and lower in the systemically healthy with chronic periodontitis.^6^ In the Canakci et al. study,^7^ higher salivary MDA levels and lower salivary SOD and GPx activities were detected in periodontitis patients compared with the healthy controls. Inflammation and oxidative stress may be effectively altered with dietary interventions, such as consuming foods and beverages rich in polyphenols.^8,9^

 Cranberry (Vaccinium macrocarpon Ait. Ericaceae) which is widely consumed in the forms of juice, fresh fruits, dry fruits, and encapsulated powders is a rich source of polyphenolic compounds including flavonoids, phenolic acids, and complex phenolic polymers with beneficial biological properties for human health.^10^ It was previously shown that the proanthocyanidins (PACs) of cranberry may be useful in treating oral infections such as dental caries. It is also suggested that cranberry may be beneficial for periodontal health.^9,11^ Though limited, it was also shown that cranberry juice is effective in reducing serum glucose in patients with diabetes.^12-14^ Wilson et al^13^ demonstrate that the consumption of a low-calorie (38 calorie/480 ml) cranberry juice rich in proanthocyanidins is associated with a favorable glycemic response and may be beneficial for persons with impaired glucose tolerance.^13^ One explanation for this effect may be a delay in the gastric uptake of glucose or distribution of glucose to insulin‐sensitive tissues following cranberry juice consumption.^14^ Furthermore, in terms of antioxidant capacity cranberry is highly ranked among polyphenol-rich beverages such as green tea and red wine.^15^ It is recognized as a rich source of quercetin, myricetin glycosides, and larger proanthocyanidin polymers.^13,16,17^ Studies have shown that quercetin can inhibit gastric glucose uptake in pigs. Additionally, both quercetin and myricetin have been found to impede glucose transporter type 4) GLUT4(-mediated glucose uptake in rat adipocytes^13,18^, as well as to inhibit aldose reductase^19^, α-amylase^20^, and α-glucosidase activities in vitro.^21^ So it can be beneficial in patients with diabetes. It also contains acetylsalicylic acid, which has anti-inflammatory properties.^22^ Some clinical studies with 2 to 16 weeks of cranberry juice consumption showed an increased plasma antioxidant capacity following the intervention in healthy subjects^22-25^ and patients with type 2 DM.^18,19^ Basu et al^26^ in an 8-week study reported a significant increase in plasma antioxidant capacity and a significant decrease in MDA, but no changes in C-reactive protein and interleukin-6 in female subjects with metabolic syndrome. In contrast to these findings, Kim et al^27^ in an experimental model showed that the mean serum levels of CRP and IL-6 were significantly lower in the cranberry powder groups compared with the normal diet group. Furthermore, Duthie et al^28^ in a 2-week interventional study showed no significant changes in blood or cellular antioxidant status following cranberry juice in healthy female subjects.

 Fish and fish oil with high unsaturated fatty acids (FAs), including n-3 series (omega-3 FAs) constituted mainly of docosahexaenoic acid (DHA) and eicosapentaenoic acid (EPA), may prevent the development of chronic inflammatory diseases through several mechanisms mainly via their anti-inflammatory activities. Topical application of these fatty acids provides considerable protection against inflammation and bone loss associated with periodontitis in experimental models.^29^

 In our previous study, we showed that cranberry juice enriched with omega-3 has beneficial effects on glycemic and periodontal status in type 2 diabetic patients with periodontal disease.^9^ Regarding the bidirectional relationship between periodontitis and type 2 diabetes mellitus, and considering the probable role of inflammation and oxidative stress in underlying the interaction between DM and periodontitis, it seems that reducing inflammatory mediators and oxidative stress may be beneficial in the treatment of periodontitis and diabetes.^30^ There are mechanistic studies supporting the anti-inflammatory and antioxidant effects of cranberries in animal models.^31,32^ However, limited clinical trials have provided evidence of the therapeutic effects of cranberries on the improvement of inflammation and oxidative stress especially in type 2 diabetics and periodontal disease. So, more investigations are needed in this area. Therefore, the present study aimed to investigate the changes in serum and salivary TAC and MDA and serum uric acid, TNF-α, IL-6, and hs-CRP and periodontal status in diabetic patients with periodontal disease following the consumption of cranberry juice, omega-3 fatty acids, and their together enrichment.

## Methods

###  Sample size

 This randomized, parallel, intervention study conformed to the ethical guidelines of the 1975 Declaration of Helsinki and was approved by the Ethics Committee of Ahvaz Jundishapur University of Medical Sciences (Ethical Code: AJUMS. REC.1392.17). The sample size was determined based on the primary information obtained from the study by Chapple IL et al^33^ for PD (Pocket depth) as the main variable. Regarding α value equal to 0.05 and a power of 80%, (α = 0.05 and β = 0.2) the sample size was computed using the appropriate formula^8^ as 9 subjects per group (4 groups were selected). Considering the withdrawal of 30%, forty-eight diabetic patients (35-67 y) with chronic adult periodontal disease were recruited from the Endocrinology Clinic of Golestan Hospital in Ahvaz city, Iran.

###  Inclusion and exclusion criteria 

 The inclusion criteria included: male or female subjects aged between 35 and 70 years old; history of at least five years diagnosed with type 2 diabetes mellitus; moderate periodontal diseases based on the probing depth (≥ 4 mm in at least one site in three-quarters of mouth) and radiographic photos^34^, CAL = 1–4 mm and BMI ranged between 18.5 to 35 kg/m^2^.

 Subjects were excluded if they had the following criteria: hospitalized due to any complications of diabetes, any diseases affect levels of glycosylated hemoglobin such as anemia, hemodialysis, hemoglobinopathies, uremia, pregnancy and lactation, travel more than 2 weeks, smoking, other serious systemic diseases, noticeable change in diet in the past six months, noticeable change in consumption of medications and treatment of diabetes, having periodontal treatment for at least 6 months, receiving immunosuppressive drugs, or any dietary supplements including antioxidant supplements.

###  Participants and interventions

 A written informed consent was obtained from all patients. By another investigator using a random-number table, subjects were randomly allocated to one of 4 groups, including one control group (C; n = 12, receiving only non-surgical periodontal treatment), and three intervention groups of I1 (n = 10), I2 (n = 9) and I3 (n = 10) receiving 1 g omega-3 fatty acid capsule twice daily, cranberry juice (200 ml, twice daily) and cranberry juice enriched with omega-3 fatty acid (200 ml, containing 1 g omega-3 fatty acid) twice daily for 8 weeks respectively. All subjects were asked to maintain their routine diet and physical activity during the study. Subjects were asked to keep the juice under refrigeration, avoid exposing the drink to direct heat or light, and avoid consuming the juices with any other snack, lunch, or dinner. Subjects were asked to bring back unconsumed juice and omega-3 supplements to assess compliance. The routine periodontal treatment was done for all patients at the beginning of the study and continued during the following weeks and after one month based on the severity of the diseases. The periodontal treatment included the education of oral and dental hygiene, using mouthwash, and scaling and root planning of teeth.

###  Cranberry juice and omega-3 supplement

Table 1 shows the nutrient and physical and chemical characteristics of the beverages used in our study. Subjects in I2 and I3 groups received either 200 ml cranberry juice or cranberry juice enriched with omega-3 fatty acid twice daily for 8 weeks. Both kinds of cranberry juices were supplied by Takdaneh Industry & Cultivate Company, Marand City, East Azerbaijan, Iran, in identical Tetra Pak cardboard packaging, each package containing 200 ml of cranberry juice or cranberry enriched with omega-3 fatty acids, kept under refrigeration at the study site. In addition, the group received 1 g omega-3 fatty acid capsule [from DSM Company, Heerlen, Netherlands, containing 180 mg eicosapentaenoic acid (EPA) and 120 mg docosahexaenoic acid (DHA) twice daily.

**Table 1:** Components and values of cranberry juice per 400 mL^a^

**Component**	**Cranberry juice**
Calories (kcal)	48.00
Sugar: Fructose (g)	5.20
Sugar: Glucose (g)	1.66
Sugar: Sucrose (g)	0.66
Ascorbic acid (mg)	92.00
Total phenolics (mg)	390.00
Total anthocyanins (mg)	16.00
Proanthocyanidins (mg)	214.00
pH	5.70
Brix (°Bx)	9.60
Haze (NTU)	42.40

^a^Participants received 400 mL of cranberry juice or cranberry juice enriched with omega-3 daily for 8 weeks. Total phenolics and anthocyanins were determined by high-performance liquid chromatography (HPLC). Kcal: kilocalorie; g: gram; mg: milligram; Bx: Brix; NTU: nephelometric turbidity unit; mL: milliliter.

###  Assessment of anthropometric indices and dietary intake 

 Body weight was measured using an analog scale (Seca, Germany) with 0.1 kg accuracy and height was measured using a stadiometer (Seca, Germany) with 0.5cm accuracy. BMI was calculated as the weight in kilograms divided by the height in meters squared. Waist and hip circumferences were measured using a tape measure with an accuracy of 0.5 cm at baseline and post-intervention. A 24-hour dietary recall of 3 days including 2 week days and one weekend was collected at baseline and post-intervention. The dietary analysis was done using Nutritionist 4 software (First Databank Inc., Hearst Corp., San Bruno, CA).

###  Assessment of biochemical parameters

 A venous blood sample (10 ml) and unstimulated saliva sample (2-3 ml) were collected from subjects after overnight fasting at baseline and end of the study and processed for biochemical analysis. Salivary and serum markers of oxidative stress such as TAC were measured by reliable spectrophotometric methods using a Randox kit (RANDOX, UK). The unsaturated lipid peroxidation of low-density lipoprotein (LDL) was evaluated by measuring the formation of thiobarbituric acid reactive substances (TBARS). TBARS were calculated as MDA equivalents using standard freshly diluted 1, 1, 3, 3- tetra methoxypropane. We assessed serum hs-CRP by the immunoturbidimetry method and serum IL-6 and TNF-α by commercial enzyme-linked immunosorbent assay (ELISA) kit (Human IL-6 and TNF-α Elisa kit [RANDOX, UK]). Serum uric acid was assessed with spectrophotometric methods using a Pars Azmun kit, in Iran.

###  Evaluation of periodontal status

 The periodontal indices including the presence or absence of BOP (bleeding on probing; bleeding that is induced by gentle manipulation of the tissue at the depth of the gingival sulcus, or interface between the gingiva and a tooth) and plaque (i.e., the measurement of the state of oral hygiene based on recording both soft debris and mineralized deposits on teeth), PD and CAL were measured by a dentist at six sites of a tooth (mesiobuccal, midbuccal, distobuccal, mesiolingual, midlingual, and distolingual). The CAL was evaluated by a full-mouth periodontal examination and determined by measuring the distance from the cement–enamel junction to the bottom of the gingival crevice. Periodontitis is defined as severe in individuals with CAL≥5 mm (not on the same tooth) moderate in individuals with CAL of 3–4 mm (not on the same tooth) or weak in individuals with CAL of 1–2 mm (not on the same tooth). PD (the distance between the gingival margin and the base of the gingival sulcus or periodontal pocket) was recorded using aUNC-15 (University of North Carolina No. 15) manual periodontal probe at six sites per tooth.^34,35^ At the beginning of the study, non-surgical periodontal treatment including scaling and root surface debridement was conducted for both intervention and control groups. Also, some instructions for dental hygiene such as how to brush and use dental floss correctly were provided. The patients were instructed to avoid consuming mouthwash.

###  Statistical analysis

 Data were analyzed using SPSS (version 16; SPSS Inc., Chicago, IL). All results were expressed as mean ± SD for quantitative data and number and frequency for quantitative data. The normal distribution of variables was tested by visual examination of the data and confirmed by Kolmogorov- Smirnov test. The baseline differences of mean values were tested using a one-way analysis of variance (One-way ANOVA). Analysis of covariance (ANCOVA) was used to identify any differences between the four groups at the end of the study, adjusting for baseline values and covariates. For the quantitative variable Chi-square test was used for comparison of mean values between groups. The mean values were compared within groups’ pre and post-intervention using paired sample t-tests. *P* < 0.05 was considered statistically significant.

## Results

###  General characteristics, anthropometric status, and energy and dietary intake

**Figure 1:**
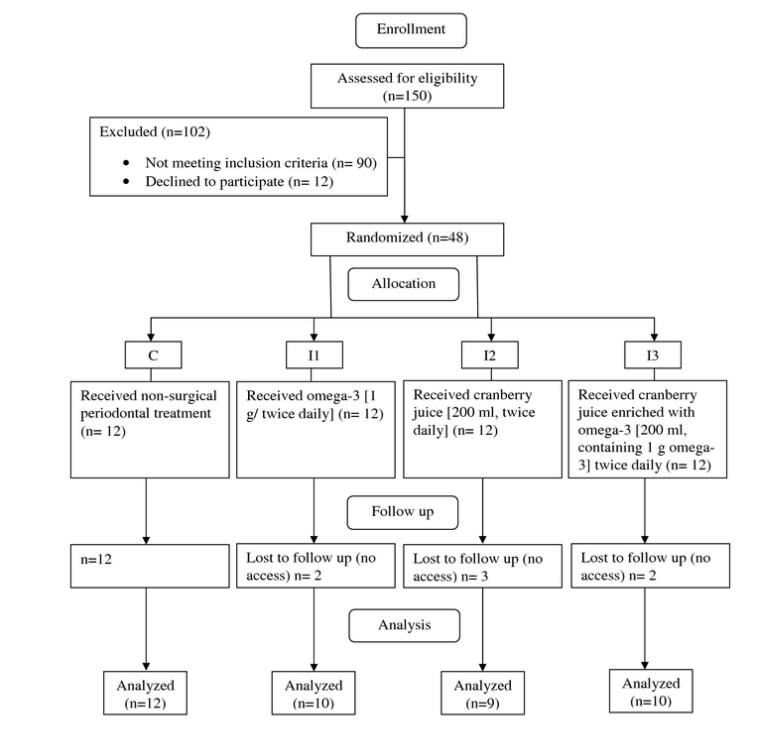
Flow diagram of the study

 Forty-one subjects were randomly allocated to one of four groups including one control group (C; n = 12), and three intervention groups of I1 (n = 10), I2 (n = 9), and I3 (n = 10) received interventions for 8 weeks and completed the study (Figure 1). Fourteen subjects (34.14%) were male and 27 (65.85%) were female. Out of 12 recruited subjects for each group, 2,3, and 2 withdrew due to lack of access to them from groups I1, I2, and I3 respectively, leading to a 12 – 25 % drop-out rate in these groups. The mean age of subjects was 55.61 ± 6.98 years old. General characteristics and anthropometric status of subjects are shown in Table 2. There were no significant differences between the four groups in age, weight, BMI, and WC at baseline and post-intervention (*P* ≥ 0.05).

**Table 2:** General characteristics and anthropometric indices of subjects at baseline and after the intervention

**Variable**	**Time**	**C (n=12)**	**I1 (n=10)**	**I2 (n=9)**	**I3 (n=10)**
Age (y)	Baseline	53.60 ± 6.23	57.75 ± 8.58	57.88 ± 6.03	53.14 ± 6.91
Male/Female (n)	Baseline	2/10	5/5	5/4	2/8
Height (cm)	Baseline	161.30 ± 7.52	164.06 ± 7.16	163.89 ± 9.59	156.64 ± 13.23
Weight (kg)	Baseline	73.60 ± 7.17	75.50 ± 12.68	73.22 ± 12.71	66.78 ± 11.76
	After intervention	74.20 ± 7.32	77.12 ± 12.28	73.27 ± 13.31	66.00 ± 11.23
BMI (kg/m^2^)	Baseline	28.38 ± 3.18	27.94 ± 3.43	27.58 ± 6.72	27.64 ± 6.92
	After intervention	28.61 ± 3.32	28.57 ± 3.40	27.65 ± 7.12	27.24 ± 6.24
Waist circumferences (cm)	Baseline	104.00 ± 10.51	105.25 ± 11.32	98.88 ± 9.43	102.71 ± 14.19
	After intervention	104.60 ± 10.45	106.25 ± 10.68	99.88 ± 10.12	102.71 ± 13.17

C: control group: I1: omega-3 group: I2: cranberry juice group: I3: cranberry juice enriched with omega-3 group; BMI: body mass index. The results are described as mean ± standard deviation (SD) Difference between groups at baseline and after the intervention (one-way ANOVA). There were no significant differences between the groups in age, weight, BMI, and WC at baseline and after the intervention (*P* ≥ 0.05).

 There were no significant differences in energy, macronutrients, and micronutrient intake between and within the four groups during the study (Table 3).

**Table 3:** Dietary intakes of subjects at baseline and after the intervention

**Variable**	**Time**	**C (n=12)**	**I1 (n=10)**	**I2 (n=9)**	**I3 (n=10)**	***P*** value^*^
Energy (kcal/day)	Baseline	1416.40 ± 190.92	1634.00 ± 238.99	1516.90 ± 172.74	1505.90 ± 240.66	0.209
	After intervention	1370.20 ± 153.54	1501.90 ± 380.53	1486.10 ± 164.16	1645.80 ± 367.01	0.262
*P* ^**^		0.144	0.243	0.538	0.470	
Carbohydrate (g/d)	Baseline	205.68 ± 24.49	228.89 ± 21.81	240.11 ± 28.92	215.51 ± 35.81	0.062
	After intervention	200.76 ± 20.66	212.56 ± 17.58	237.64 ± 30.55	243.72 ± 54.65	0.071
*P* ^**^		0.169	0.147	0.587	0.273	
Protein (g/d)	Baseline	57.21 ± 13.94	64.55 ± 11.39	56.96 ± 9.60	63.58 ± 16.22	0.485
	After intervention	54.39 ± 13.29	55.02 ± 12.07	57.15 ± 8.70	68.12 ± 20.30	0.251
*P* ^**^		0.104	0.066	0.949	0.676	
Total Fat (g/d)	Baseline	41.35 ± 9.14	48.22 ± 17.59	37.88 ± 8.50	43.61 ± 12.57	0.377
	After intervention	39.82 ± 6.41	36.24 ± 7.12	35.83 ± 9.96	45.90 ± 14.92	0.241
*P* ^**^		0.354	0.096	0.625	0.778	
Dietary fiber (g/d)	Baseline	13.83 ± 6.12	12.55 ± 4.93	13.21 ± 4.42	10.90 ± 3.88	0.647
	After intervention	13.52 ± 6.04	11.61 ± 4.97	11.79 ± 2.47	13.33 ± 6.56	0.794
*P* ^**^		0.577	0.344	0.284	0.347	
Vitamin A (μg/d)	Baseline	230.60 ± 178.05	465.10 ± 176.50	461.10 ± 357.07	632.40 ± 618.00	0.070
	After intervention	233.20 ± 187.30	249.80 ± 159.70	312.20 ± 155.80	488.10 ± 281.10	0.080
*P* ^**^		0.500	0.060	0.069	0.060	
Vitamin E (mg/d)	Baseline	49.27 ± 39.24	42.50 ± 43.10	19.80 ± 43.10	632.40 ± 618	0.059
	After intervention	48.80 ± 38.20	42.90 ± 37	40.2 ± 69.40	12.60 ± 4.90	0.680
*P* ^**^		0.700	0.900	0.300	0.900	
Vitamin C (mg/d)	Baseline	56.26 ± 58.90	50.08 ± 10.80	70.40 ± 60.60	54.60 ± 34.40	0.480
	After intervention	53.30 ± 58.10	39.90 ± 15.90	51.30 ± 23.70	57.06 ± 47.10	0.180
*P* ^**^		0.350	0.060	0.190	0.880	

C: control group; I1: omega-3 group; I2: cranberry juice group; I3: cranberry juice enriched with omega-3 group The results are described as mean ± standard deviation (SD). *Difference between groups at baseline and after the intervention (one-way ANOVA). **within group difference (paired t-test) *P* < 0.05 was considered significant.

###  Serum hs -CRP, IL-6, and TNF-α

 The mean levels of serum hs-CRP, IL-6, and TNF-α did not significantly differ between the four groups at baseline (Table 4). The mean of hs-CRP and IL-6 levels was significantly reduced post-intervention in cranberry enriched with omega-3 group (0.97 ± 0.66; *P* = 0.003) and (1.22 ± 0.19; *P* = 0.02) respectively, compared to the control group (1.73 ± 0.46) and (1.9 ± 0.9). Furthermore, within-group comparison in four groups showed that the mean levels of hs-CRP (*P* = 0.008), IL-6 (*P* = 0.005), and TNF-α (*P* = 0.04) were reduced compared with the baseline. However, it was significant only in cranberry enriched with omega-3 group.

**Table 4:** Serum levels of IL-6 and Hs-CRP of subjects at baseline and after the intervention

**Variable**	**Time**	**C (n=12)**	**I1 (n=10)**	**I2 (n=9)**	**I3 (n=10)**	***P*** ** value**
Hs-CRP (mg/dL)	Baseline	1.87 ± 2.40	1.92 ± 3.23	1.84 ± 5.10	1.31 ± 0.72	0.890^a^
	After intervention	1.73 ± 0.46^d^	1.67 ± 2.59	1.62 ± 2.60	0.97 ± 0.66^c^	0.003^b^
	Change	-0.14 ± 2.28	-0.25 ± 0.78	-0.22 ± 2.60	-0.34 ± 0.49	0.570^b^
*P* ^c^		0.280	0.320	0.540	0.008	
IL-6 (ng/mL)	Baseline	2.14 ± 1.00	2.42 ± 1.98	2.05 ± 1.40	2.32 ± 0.40	0.320^a^
	After intervention	1.90 ± 0.90^d^	1.45 ± 1.10	1.42 ± 1.30	1.22 ± 0.190^c^	0.020^b^
	Change	-0.24 ± 1.20	-0.97 ± 1.05	-0.63 ± 1.60	-1.10 ± 0.50	0.690^b^
*P* ^c^		0.500	0.440	0.810	0.005	
TNF-α (ng/ mL)	Baseline	9.12 ± 3.87	9.07 ± 3.58	8.78 ± 2.45	9.15 ± 2.63	0.620
	After intervention	9.01 ± 3.95	8.94 ± 3.45	8.66 ± 3.09	8.85 ± 2.79	0.530
	Change	-0.11 ± 3.91	-0.13 ± 3.10	-0.12 ± 2.67	0.30 ± 2.71	0.130
*P* ^c^		0.200	0.380	0.740	0.040	

C: control group; I1: omega-3 group; I2: cranberry juice group; I3: cranberry juice enriched with omega-3 group hs-CRP: high-sensitivity C-reactive protein; IL-6: interkeukin-6; TNF-α: tumor necrosis factor-alpha The results are described as mean ± standard deviation (SD) ^a^ Difference between groups at baseline, the *P* value is reported based on one-way ANOVA. ^b^ Difference between groups post-intervention; the *P *value is reported based on analysis of covariance (ANCOVA). ^c^ Intra-group differences; the *P *value is reported based on the paired t-test. *P*< 0.05 was considered significant.

###  Serum TAC, MDA and uric acid 

 There were no significant differences observed between the four groups in the mean serum TAC at baseline (Table 5). Adjusting for baseline values of BMI and energy intake, the analysis of covariance showed a significant difference in the mean serum TAC between four groups post-intervention (*P* = 0.04). Moreover, the Pair pair-wise comparisons showed that there were significant differences in serum TAC between the I3 group (0.31 ± 0.39 mmol/l) and the control group (-0.2 ± 0.16 mg/dl) post-intervention. It was found that the mean level of serum TAC in the I3 group was 0.34 mmol/l higher compared with the control group.

**Table 5:** Serum TAC, MDA, and UA of subjects at baseline and after the intervention

**Variable**	**Time**	**C (n=12)**	**I1 (n=10)**	**I2 (n=9)**	**I3 (n=10)**	***P *** **value**
TAC (mg/L)	Baseline	1.23 ± 0.29	1.32 ± 0.42	1.33 ± 0.28	1.29 ± 0.36	0.900^a^
	After intervention	1.21 ± 0.28^e^	1.47 ± 0.43	1.39 ± 0.31	1.60 ± 0.23^d^	0.040^b^
	Change	-0.20 ± 0.16	0.14 ± 0.43	0.05 ± 0.31	0.31 ± 0.39	0.160^b^
*P* ^c^		0.610	0.310	0.560	0.043	
UA (mg/dL)	Baseline	5.37 ± 1.70	5.90 ± 1.80	5.10 ± 1.20	5.70 ± 1.68	0.700^a^
	After intervention	5.50 ± 1.70	6.40 ± 2.20	5.70 ± 1.34	6.30 ± 1.58	0.680^b^
	Change	0.20 ± 0.61	0.51 ± 1.10	0.78 ± 0.93	0.56 ± 0.84	0.700^b^
*P* ^c^		0.280	0.180	0.600	0.070	
MDA (mmol/L)	Baseline	3.70 ± 1.30	4.90 ± 2.01	4.60 ± 1.30	4.50 ± 2.10	0.400^a^
	After intervention	3.15 ± 1.67^e^	3.96 ± 1.2	2.90 ± 1.20	2.70 ± 1.40	0.150^b^
	Change	-0.47 ± 1.40^h^	-1.20 ± 1.70	-1.60 ± 0.61^g^	-2.01 ± 1.68^f^	0.040^b^
*P* ^c^		0.310	0.080	< 0.001	0.010	

C: control group; I1: omega-3 group; I2: cranberry juice group; I3: cranberry juice enriched with omega-3 group; TAC: total antioxidant capacity; MDA: malondialdehyde; UA: uric acid The results are described as mean ± standard deviation (SD) ^a^ Difference between groups at baseline value is reported based on one-way ANOVA. ^b^ Difference between groups after the intervention; the *P* value is reported based on analysis of covariance (ANCOVA). ^c^ Intra-group difference; the *P* value is reported based on the paired t-test. *P* < 0.05 was considered significant. Pairwise significant results (LSD): d vs e. Pairwise significant results (LSD): f and g vs. h.

 No significant difference was found in serum uric acid between and within groups (Table 5). Regarding serum MDA, there were no significant differences observed between the four groups pre and post-intervention. However, the mean change of MDA significantly differed between the four groups post-intervention (*P* = 0.04). The PairWise Comparisons showed that the mean changes of MDA in the I2 group (-1.6 ± 0.61 µmol/l) and I3 group (-2.01 ± 1.68 nmol/ml) were significantly differed compared with the control group (-0.47 ± 1.4 nmol/ml). The mean change of serum MDA in intervention groups of I2 and I3 was decreased by 1.7 µmol/l and 1.3 nmol/ml respectively compared with the control group. Moreover, serum MDA was significantly decreased in intervention groups of I2 (*P* < 0.001) and I3 (*P*= 0.01) compared to its baseline (Table 5).

###  Salivary TAC and MDA 

 There was no significant difference in the mean TAC of salvia between the four groups at baseline and post-intervention (Table 6). According to Pair Wise Comparisons, a significant increase was observed in the TAC of salvia in the I3 group (1.1 ± 0/63 mg/dl) compared with the control group (0.48 ± 0.29 mmol/l). In other words, the TAC of saliva in the I3 group was approximately 0.47mmol/l greater than the control group.

**Table 6:** Salivary levels of TAC and MDA of subjects at baseline and after the intervention

**Variable**	**Time**	**C (n=12)**	**I1 (n=10)**	**I2 (n=9)**	**I3 (n=10)**	***P*** ** value**
TAC (mg/dL)	Baseline	0.47 ± 0.22	0.77 ± 0.34	0.61 ± 0.3	0.91 ± 0.74	0.120^a^
	After intervention	0.48 ± 0.29^e^	0.79 ± 0.43	0.13 ± 0.51	1.10 ± 0.62^d^	0.120^b^
	Change	0.00 ± 0.29	0.02 ± 0.36	0.13 ± 0.51	0.2 ± 0.92	0.790^b^
*P* ^c^		0.690	0.880	0.430	0.530	
MDA (mmol/L)	Baseline	0.45 ± 0.23	0.83 ± 0.45	0.54 ± 0.29	0.82 ± 0.67	0.100^a^
	After intervention	0.59 ± 0.40^e^	0.71 ± 0.30	0.49 ± 0.21	0.55 ± 0.39^d^	0.530^b^
	Change	0.14 ± 0.40	-0.12 ± 0.44	-0.04 ± 0.30	-0.27 ± 0.49	0.160^b^
*P* ^c^		0.250	0.400	0.650	0.130	

C: control group: I1: omega-3 group: I2: cranberry juice group: I3: cranberry juice enriched with omega-3 group: TAC: total antioxidant capacity; MDA: malondialdehyde The results are described as mean ± standard deviation (SD) ^a^ Difference between groups at baseline; the *P* value is reported based on one-way ANOVA. ^b^ Difference between groups after the intervention; the *P* value is reported based on analysis of covariance (ANCOVA). ^c^ Intra-group difference; the P-value is reported based on the paired t-test. *P* < 0.05 was considered significant. Pairwise significant results (LSD): d vs. e.

 The mean MDA of salvia (Table 6) did not significantly differ between the four groups pre and post-intervention. Salivary MDA level was reduced (but not significantly) in four groups post-intervention compared with its baseline. A significant decrease was observed in the MDA of salvia in the I3 group (-0.27 ± 0.49 nmol/ml) compared with the control group (0.14 ± 0.4 mg/dl).

###  Periodontal indices

 The mean change of PD and CAL had a significant difference between the four groups post-intervention (*P* = 0.03 and *P* = 0.007 respectively). The Pairwise Comparisons showed that the mean changes in the omega-3 fatty acid group (-1.08 ± 0.49 mm) were higher compared with the cranberry juice group (-0.56 ± 0.3 mm). In addition, based on pairwise comparisons the mean changes in cranberry juice enriched with omega-3 fatty acid group (1.38 ± 0.21) were higher compared with the control group (1.69 ± 0.65). Moreover, PD and CAL were significantly reduced in four groups after 8 weeks compared to the baseline (*P* < 0.001). No significant difference was observed for Plaque and BOP within and between groups (*P* > 0.05) (Table 7).

**Table 7:** Periodontal status at baseline and after the intervention

**Variable**	**Time**	**C (n=12)**	**I1 (n=10)**	**I2 (n=9)**	**I3 (n=10)**	***P*** value^*^
PD (mm)	Baseline	2.42 ± 0.50	2.50 ± 0.61	2.06 ± 0.54	2.36 ± 0.41	0.220^a^
	After intervention	1.50 ± 0.45	1.40 ± 0.28	1.49 ± 0.33	1.59 ± 0.31	0.310^b^
	Change	-0.90 ± 0.49	-1.08 ± 0.49^c^	-0.56 ± 0.3^d^	-0.77 ± 0.3	0.030^b^
*P* ^c^		< 0.001	< 0.001	< 0.001	< 0.001	
CAL (mm)	Baseline	3.22 ± 0.35	3.40 ± 0.51	3.31 ± 0.24	3.56 ± 0.81	0.830^a^
	After intervention	1.69 ± 0.65^d^	1.53 ± 0.32	1.47 ± 0.33	1.38 ± 0.21^c^	0.050^b^
	Change	-1.53 ± 0.50^d^	-1.87 ± 0.42	-1.84 ± 0.28	-2.18 ± 0.51^c^	0.007^b^
*P* ^c^		< 0.001	< 0.001	< 0.001	< 0.001	
Plaque ( + )	Baseline (N, %)	12 (100)	10 (100)	9 (100)	10 (100)	0.430^e^
	After intervention	7 (58.30)	5 (50%)	4 (44.40)	4 (40)	
	Change	4	5	5	6	
BOP ( + )	Baseline (N, %)	12 (100)	10 (100)	9 (100)	10 (100)	0.170^e^
	After intervention	8 (66.60)	6 (60%)	5 (55.50)	5 (50)	
	Change	5	4	4	5	

C: control group; I1: omega-3 group; I2: cranberry juice group; I3: cranberry juice enriched with omega-3 group PD: pocket depth; CAL: clinical attachment loss; BOP: bleeding on probing The results are described as mean ± standard deviation (SD). ^a^ Difference between groups at baseline; the *P* value is reported based on one-way ANOVA. ^b^ Difference between groups after the intervention; the P-value is reported based on analysis of covariance (ANCOVA). ^c^ Intra-group difference; the *P *value is reported based on the paired t-test. ^e^ Difference between the groups using the chi-squared test. *P* < 0.05 was considered significant. Pairwise significant results (LSD): c vs. d.

## Discussion

 In the present study, the anti-inflammatory and anti-oxidative effects of cranberry juice enriched with omega-3 fatty acids were investigated in patients with diabetes and periodontal disease. Based on the studies the consumption of a low-calorie cranberry juice not only is not prohibited on a diabetes diet, but it also has significant beneficial effects on blood sugar, thereby can control oxidative stress and inflammatory reactions in patients with diabetes.^12-15^ It is proposed that the host’s inflammatory response, an ongoing cytokine-induced acute-phase response, is closely involved in the pathogenesis of type 2 diabetes and periodontal disease.^36^ The activation of inflammation at a systemic level can result in the chronic elevation of inflammatory mediators including IL-1, TNF-α, IL-6, and PGE2, and acute phase reactants such as C-reactive protein, elevated fibrinogen, and lowered albumin, which all are hallmarks of the acute phase reaction (APR) that are observed in diabetes and periodontitis.^37^ Therefore it is suggested that the risk of periodontal disease may be reduced through the effective control of metabolic status in diabetic patients and vice versa the treatment of periodontal disease, which may be accompanied by the reduction of inflammatory markers and oxidative stress, may improve the insulin sensitivity in diabetic patients with periodontal disease.^38^

 It is found that several functional foods and nutritional supplements with anti-inflammatory and anti-oxidative properties in adjunct with non-surgical periodontal treatment (NSTs) may be important factors affecting periodontal disease.^39^

 To our knowledge, this was the first study that evaluated the anti-inflammatory and anti-oxidative effects of cranberry juice alone or accompanied by omega-3 fatty acid in adjunct with nonsurgical periodontal treatment in patients with diabetes and periodontal disease. In the present study serum levels of IL-6 and hs-CRP were significantly decreased in the group receiving cranberry juice enriched with Omega-3 fatty acids compared to the control group. Clinical trials have reported conflicting results on the effects of polyphenol supplementation on biomarkers of inflammation. Karlsen et al^40^ in a short-term, 4-week trial in subjects at increased risk of CVD, showed that bilberry juice supplementation decreased plasma CRP and IL-6, while green tea supplementation for 8 weeks showed no effects on these inflammatory parameters in subjects with metabolic syndrome.^41^ Our finding was also in contrast with the results of an 8-week interventional study by Basu et al^26^ that showed no significant effects of cranberry juice intervention on interleukin-6 (IL-6) and C-reactive protein (CRP) in subjects with metabolic syndrome. Tipton et al^42^ in an *in-vitro *study showed that cranberry components inhibit IL-6 and other inflammatory markers production by human gingival epithelial cells and fibroblasts which concurs with the findings of the present study. Also, an *in-vitro* study Galarraga-Vinueza et al^43^ demonstrated that Pro-inflammatory cytokine expression (ie IL-8 and IL-6) was downregulated in LPS-stimulated macrophages by cranberry concentrates at 50 and 100 µg/mL. Similarly, Xue et al^30^ in an in-vitro study showed that phytochemicals present in varying quantities in cranberry fruits including anthocyanins, hyperoside, ursolic acid, and corosolic acid play a role in the anti-inﬂammatory eﬀects of cranberry extracts. In their study, eight extracts from cranberry fruits were prepared, analyzed for phytochemical composition, and evaluated for their anti-inﬂammatory eﬀects in human monocytes (THP-1 cells). The extracts varied widely in polyphenol and triterpenoid content. All were able to reduce lipopolysaccharide (LPS)-induced production of pro-inﬂammatory cytokine interleukin 6 (IL-6) at 100 μg/mL, with inhibition ranging between 18.8 and 48.8%. Of these, three extracts high in anthocyanins, triterpenoids, or total polyphenols decreased levels of IL-6 and tumor necrosis factor-alpha (TNF-α) at concentrations of 0.1−10 μg/mL compared to LPS-exposed control. Several individual cranberry phytochemicals were also capable of reducing the production of IL-6, IL-1β, and TNF-α. Consistent with the results of our study, an animal study by Cai et al^44^ reported that colonic levels of pro-inflammatory cytokines (IL-1β, IL-6, and TNF-α) were significantly reduced by cranberry supplementation. According to some studies, serum levels of IL-6 are higher in patients with periodontal disease than in healthy controls. Gingival epithelial cells and fibroblasts may produce IL-6 as a regulator of osteoclast bone resorption due to the destruction of gum tissue, which is involved in periodontal inflammation.^45,46^

 Many dental researchers have indicated that periodontal infection-related TNF-α contributes to systemic inflammatory reactions that can impair insulin signaling by increasing the adipose tissue secretion of free fatty acids^47^ and raising insulin resistance. This hypothesis suggests that periodontal therapy can effectively improve glycemic control in diabetic patients by decreasing proinflammatory mediators.^38^ In the present study, besides IL-6 and hs-CRP, the mean levels of TNF-α decreased in all groups, but it was significant only in the group receiving cranberry juice enriched with omega-3 fatty acids. It was found that cranberry, a rich source of several bioactive flavonoids, may affect periodontal bacteria and inhibit tissue destruction mediated by bacterial proteinases.^48^ It is suggested that proanthocyanidins, the high molecular weight components in cranberry, may reduce the production of lipopolysaccharide-induced inflammatory markers from gingival epithelial cells and fibroblasts through the inhibition of nuclear factor kappa B (NF-κB), matrix metalloproteinases (MMP-1, MMP-2, and MMP-3), and activator protein 1 (AP-1), which is an important transcription factor for the genes coding for pro-inflammatory mediators. Therefore, they may be useful in the host response and treatment of periodontal disease.^49^ The diversity of results in studies may be attributed to the dose of cranberry or differences in study design, subject characteristics, and lifestyle.

 Several studies have indicated that the consumption of omega-3 fatty acids, specifically EPA and DHA, which are known as anti-inflammatory components^50^, is inversely associated with periodontitis.^51,52^ The findings of the present study suggest a possible synergistic effect of omega-3 fatty acids and the bioactive components of cranberry juice on inflammatory markers.

 The imbalance between the production of reactive oxygen species (ROS) and antioxidant defenses, leading to oxidative stress, is an important factor involved in the development of periodontal disease and DM.^5^ There is evidence showing a bilateral relationship between antioxidant capacity and periodontal disease.^53^ It has been shown that plasma antioxidant capacity decreases in patients with periodontal disease.^54^ Similarly, an inverse association was observed between antioxidant capacity and the severity of periodontal disease, according to the study by Chapple et al.^55^ In addition, it was found that NST has a positive impact on reducing the total oxidative status in periodontal patients.^56^ Therefore, it is suggested that the consumption of foods with high antioxidant content, along with NST, may be useful in alleviating inflammatory diseases.^8^ Cranberry is considered a rich source of phenolic active ingredients. Several studies have shown the beneficial effects of cranberry on oral health and periodontal disease.^10,11,49,57,58^ In the present study, receiving cranberry juice enriched with omega-3 fatty acids for 8 weeks resulted in a significant increase in serum TAC compared with the control group. Our findings are consistent with studies with intervention durations of 2 to 16 weeks, showing that cranberry juice consumption may improve oxidative stress in healthy subjects^23,25^, and patients with type 2 diabetes.^18,26^ However, our findings are inconsistent with the reports of a 2-week placebo-controlled trial by Duthie et al.^28^ This diversity in the findings may be attributed to the shorter duration of the intervention and the selection of healthy subjects in the aforementioned study.

 According to the findings of a study, lipid peroxidation, MDA levels, and oxidative stress were increased in patients with periodontal disease.^59^ In the present study, consumption of cranberry juice enriched with omega-3 fatty acids resulted in a significant decrease in serum MDA. As there was no significant difference in the intake of micronutrients within and between groups, it is suggested that the reduction in MDA may be linked to the antioxidant content of cranberry juice. Similarly, several other studies have shown that serum levels of MDA were significantly decreased in healthy individuals^24,25^, and patients with metabolic syndrome^26^ following the consumption of cranberry juice. Cranberry juice provides a higher quality of antioxidants than cranberry fruit.^15^ The ingredients in cranberry juice block and likely alleviate oxidative stress, primarily by indirectly lowering postprandial glucose and triglyceride levels—both of which contribute to oxidative stress—while also exerting a direct antioxidant effect *in vivo*.^15^

 According to some studies, salivary antioxidant levels, including uric acid, albumin, and ascorbic acid, are reduced in patients with periodontal disease. An imbalance between antioxidants and oxidative stress and the production of ROS in periodontal disease may cause this reduction.^60,61^ Mathur et al^62^ found that daily administration of 6 mg of antioxidants (2000 mcg lycopene, 7.5 mg zinc, and 35 mcg of selenium), alone or as an adjunct with NSTs for two weeks, resulted in a significant increase in salivary levels of uric acid. In the present study, uric acid levels were measured only in the serum and did not change significantly. Therefore, in future studies, it is recommended to measure the serum level of uric acid in addition to its levels in saliva.

 Several studies have shown a significant decrease in salivary TAC^63^ and a significant increase in salivary MDA, which may represent peroxidation in patients with periodontal diseases.^64,65^ In the current study, the consumption of cranberry juice enriched with omega-3 fatty acids resulted in a significant increase in TAC and a significant decrease in salivary MDA. It is suggested that this effect may result from the anti-oxidative properties of cranberry. To the best of our knowledge, there are no human studies on the effects of cranberry consumption on salivary antioxidant levels in patients with periodontitis. Therefore, further clinical trials are needed to confirm the effects of cranberry on salivary markers of oxidative stress.

 In the present study, PD and CAL were significantly reduced in all four groups after 8 weeks compared to the baseline. These results are consistent with previous studies that indicated non-surgical periodontal therapy effectively improves periodontal indices in patients with diabetes by reducing inflammatory mediators, including IL-6, TNF-α, and hs-CRP.^66-69^ Similar to the findings of these studies, inflammatory marker levels decreased in all groups in the present study. However, it was significant only in the omega-3 fatty acid and cranberry juice enriched with omega-3 fatty acid groups. Moreover, the reduction in PD in the omega-3 fatty acid group was higher than in the cranberry juice group. In addition, the reduction in the mean CAL in the cranberry juice enriched with the omega-3 group was higher than in the control group. This study is consistent with those that have pointed out the positive effect of nutritional intervention as an adjunct to non-surgical periodontal treatment in controlling periodontal diseases.^33,51,52,57,70^ Feghali et al^57^ indicated that cranberry proanthocyanidins (PACs) have a potential effect on improving periodontal diseases through various mechanisms, including the inhibition of bacterial and host-derived proteolytic enzymes, the host inflammatory response, and osteoclast differentiation and activity. In the Sharkawi et al study,^70^ daily consumptions of 900 mg fish oil (DHA and EPA) and 81 mg aspirin along with non-surgical periodontal treatment was associated with a significant reduction in pocket depth compared to the control group that received only non-surgical treatment of periodontal. Several studies also have shown that omega-3 fatty acids consumption especially EPA and DHA have inversely associated with periodontitis^51,52^ that may be due to its anti-inflammatory effects.

 In the present study, all subjects had low daily dietary fiber and other nutrient intake. Generally, higher fiber intakes are associated with more nutrient-dense diets.^71^ In addition, there were no intakes of berries, except for the cranberry juice provided during the study. Therefore, it is suggested that the effects of cranberry juice observed in our study may have been more pronounced in our subjects with inadequate dietary nutrient intakes. In the present study, the compliance of subjects (who completed the study) for drinking juices and consuming omega-3 fatty acids was 100%. Additionally, there were no reports from subjects regarding any adverse effects or symptoms with all types of intervention provided during the study. Certain limitations of the present study include a small sample size, so our findings cannot be generalized to other populations, and the short study duration. Inconsistent results of the present study with other studies may be due to differences in duration, sample size, and characteristics of participants in the present and mentioned studies. One another limitation of the present study was the sugar content (7.5 gr/400 ml) of the juices. However, it should be mentioned that this amount of sugar is very low and cranberry juice consumed in the present study is considered a low-calorie juice (48 kcal/400 ml) that is not prohibited on a diabetes diet. Because useful ingredients found in cranberry juice can delay glucose uptake and prevent the increase of blood sugar. However, it is far better and more useful to provide fruit juices without sugar or artificial sweetenerscontent. Consultation with the industry is suggested for the production of these products.

## Conclusion

 In conclusion, the consumption of cranberry juice enriched with n-3 PUFA as a nutritional approach in adjunct with non-surgical periodontal therapy may help to improve periodontal status, some salivary and serum inflammatory mediators (IL-6 and hs-CRP) and oxidative stress markers (MDA and TAC) in diabetic patients with periodontal disease. This may support the hypothesis that cranberries have anti-inflammatory and anti-oxidative effects. These findings need further investigation in larger trials with careful design, including optimal dose and form of cranberry intervention, study duration, and subject characteristics.

## Acknowledgments

 The authors express their thanks to “The Nutrition and Metabolic Disorders Research Center,” “The Research Center for Diabetes, Endocrinology, and Metabolism Clinic employees of Ahvaz University Golestan Hospital” and “The Dental Clinic of Ahvaz Jundishapur University of Medical Sciences.” We also thank all the patients for their participation in this study. This study was part of the MSc thesis of Elnaz Ashrafzadeh (NRC-9202).

## Competing Interests

 The authors declared no potential conflicts of interest.

## Consent for Publication

 Not applicable.

## Data Availability Statement

 The datasets collected and/or analyzed in the present study are not publicly accessible due to ethical concerns but the corresponding author may provide datasets upon reasonable request.

## Ethical Approval

 All participants provided written informed consent and the study was approved by the Ahvaz Jundishapur University of Medical Sciences Ethics Committee (Ethical Code: AJUMS. REC.1392.17).
